# Selective Enzymatic Esterification of Lignin-Derived Phenolics for the Synthesis of Lipophilic Antioxidants

**DOI:** 10.3390/antiox12030657

**Published:** 2023-03-07

**Authors:** Marta Martinez-Garcia, Jaime Gracia-Vitoria, Karolien Vanbroekhoven, Winnie Dejonghe, Yamini Satyawali

**Affiliations:** Separation and Conversion Technology, Flemish Institute for Technological Research (VITO), Boeretang 200, 2400 Mol, Belgium

**Keywords:** antioxidant, lignin, monolignol, enzymatic esterification, lipase

## Abstract

Lignin is an abundant and renewable source of phenolic compounds that can be used as natural antioxidants to substitute synthetic, petroleum-based alternatives. The development of lignin depolymerization techniques has improved the accessibility of low-molecular-weight phenolic fractions with enhanced antioxidant activity compared to native lignin. The selective esterification of the aliphatic OH groups in these compounds is necessary in order to increase their compatibility with hydrophobic product matrixes, while preserving their antioxidant capacity. In the present work, lipase was chosen as a selective catalyst for the esterification of the monolignol dihydroconiferyl alcohol (DCA), in order to target the esterification of aliphatic OHs without modifying the aromatic groups. The reaction was studied under solvent-assisted and solvent-free conditions, using different fatty acids and substrate ratios. A product yield of 97% could be obtained after 24 h in a solvent-assisted reaction with 2 molar equivalents of fatty acid, or after 3 h in a solvent-free reaction with 10 molar equivalents of the fatty acid. The esterified monolignol showed relevant long-term radical scavenging activity, comparable to other commercial, petroleum-based antioxidants. Different lignin fractions were also used as substrates for enzymatic esterification with different fatty acids, resulting in esterification degrees of 20–58% (of the total aliphatic OH), depending on the specific combination of fatty acid–lignin fractions.

## 1. Introduction

Antioxidants are molecules that can inhibit or delay oxidizing chain reactions via different methods (e.g., direct scavenging of reactive oxygen species, chelation of metal ions or inhibition of certain enzymes). Phenolic compounds are well-known antioxidants and their potent activity is related to the ability of the aromatic ring to donate the H from the phenolic -OH to a radical and convert itself into a stable phenoxy radical. The presence of other electron-donating or electron-withdrawing groups can contribute to its stabilization, increasing its antioxidant capacity [1].

Many natural phenolic compounds sourced from plants have been applied as antioxidants in food, cosmetics or pharmaceutical products, such as phenolic acids (e.g., gallic acid and ferulic acid), vitamins (e.g., α-tocopherol), flavonoids (e.g., catechin) or phenylethanoids (e.g., hydroxytyrosol). However, some of the most commercially used antioxidants are still petroleum-derived hindered phenols, such as butylated hydroxytoluene (BHT) and butylated hydroxyanisole (BHA) [2].

A still underexploited source of renewable phenolic compounds is lignin, a complex aromatic polymer present in the cell wall of plants. Lignin is made up of the following three monomers (known as monolignols), which differ in the number of methoxy groups in the phenolic ring: *p*-coumaryl alcohol (H- unit, no methoxy group), coniferyl alcohol (G-unit, 1 methoxy group) and sinapyl alcohol (S-unit, 2 methoxy groups). Because of these methoxy groups, monolignols have an inherent hindered phenolic structure that allows them to quench free radicals and form stabilized radicals via a similar mechanism to that of BHT or BHA [3]. The effectiveness of lignin as an antioxidant has been reported in the literature numerous times. Native or depolymerized lignin has been used to increase the thermo-oxidative stability of polymers [4,5,6,7,8], lubricant oils [9] or biofuels [10,11]. Its application in high-end products such as cosmetics is less explored and mainly focuses on the field of broad-spectrum sunscreens, where the multifunctional structure of lignin can fulfill different roles as a UV-blocker, antioxidant and antimicrobial preservative [12].

**Figure 1:**
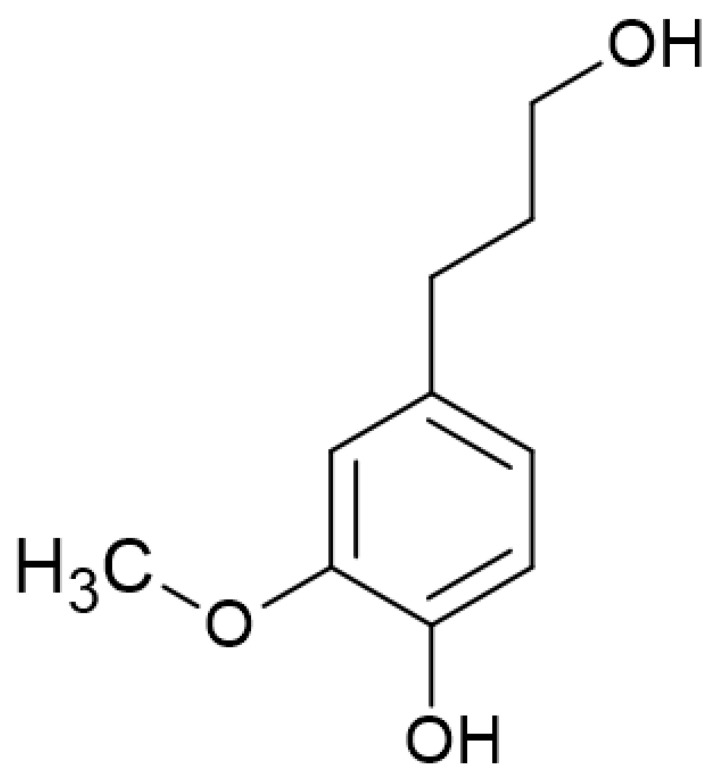
Chemical structure of the lignin-derived monomer dihydroconiferyl alcohol (DCA).

The performance of lignin as antioxidant additive can be enhanced by decreasing its molecular weight (Mw) and increasing the number of free phenolic -OHs [9,13]. The development of “lignin first” biorefinery technologies, such as reductive catalytic fractionation (RCF), has enabled the depolymerization of lignin into a low Mw oil that contains phenolic oligomers, dimers and monomers in a close-to-theoretical yield [14]. Depending on the lignin source and process conditions, the composition of monomers can change [15,16]. Dihydroconiferyl alcohol (DCA, also known as 4-propanolguaiacol) (Figure 1) is the major monomer formed by RCF of softwood lignin (e.g., pine), using Pd/C as a catalyst [17], but it is also obtained in significant quantities with hardwood lignin (e.g., birch) [15].

For their application as antioxidant additives in hydrophobic matrixes such as cosmetics, oils or polymers, lignin-derived phenols need to be lipophilized to ensure good compatibility. This can be achieved by esterifying aliphatic chains into some of the hydroxyl groups, preferably preserving the aromatic -OHs responsible for their antioxidant functionality. Chemical esterification is not selective enough and leads to the derivatization of some of the phenolic -OHs, decreasing the antioxidant capacity of the esterified lignin product [7,8]. On the contrary, enzymatic catalysis with a lipase has been shown to be selective for aliphatic -OH groups in the esterification of lignin model compounds [18] and phenyl ethanoids and propanoids [19,20,21].

In the present work, we opt for a lipase as a selective catalyst to target only the aliphatic -OH during the esterification of the lignin monomer DCA with different fatty acids, in order to obtain a lignin-based lipophilic antioxidant. The reaction was optimized to maximize the product yield and the antioxidant activity of the product was compared to other commercial antioxidants. Moreover, different lignin fractions obtained by lignin depolymerization, varying in their Mw distribution and aliphatic -OH content, were also used as substrates for enzymatic esterification. To the best of our knowledge, this is the first study that reports lipase-catalyzed esterification of depolymerized lignin fractions for application as antioxidants.

## 2. Materials and Methods

### 2.1. Chemicals

Dihydroconiferyl alcohol (DCA, 98%), octanoic acid (≥98%), decanoic acid (≥98%), lauric acid (for synthesis), *tert*-butanol (≥99%), propyl gallate (PG, ≥98%), ethyl acetate (≥99.5%), acetonitrile (≥99.9%), 2-Chloro-4,4,5,5-tetramethyl-1,3,2-dioxaphospholane (TMDP), *N*-Hydroxy-5-norbornene-2,3-dicarboxylic acid imide (NHND, 97%), 5% Pd/C and 2,2-diphenyl-1-picrylhydrazyl (DPPH) radicals were purchased from Sigma-Aldrich. (Hoeilaart, Belgium). Immobilized lipase B from *C. antarctica* (Novozym^®^ 435) was a gift from Novozymes (Bagsvaerd, Denmark). Molecular sieves (3 Å, 1.6–2.5 mm, technical, water absorption capacity 0.2 g/g) were acquired at VWR. Hexanoic acid (98%) and silica gel 60 (42–60 µm, 230–400 mesh) were purchased from Merck (Hoeilaart, Belgium). Petroleum ether (b.p = 40–60 °C), butylated hydroxytoluene (BHT, 99.8%) and butylated hydroxyanisole (BHA, 98%) were purchased from Acros Organics (Geel, Belgium).

Technical organosolv lignins were supplied by CIMV (botanical origin: wheat straw) and Fraunhofer (botanical origin: beech wood). These lignins were depolymerized as described in Section 2.6.

### 2.2. Enzymatic Esterification of DCA

Before use, molecular sieves were activated by incubation at 250 °C for at least 6 h and the reaction solvent *tert*-butanol was dried over activated molecular sieves (10% *w*/*w*) for at least 72 h. For reactions with equimolar concentration of both substrates, DCA was mixed with the fatty acid (hexanoic-, octanoic-, decanoic- or lauric acid) and *tert*-butanol (1.5 g) to give a final concentration of 50, 100, 500 or 1000 mmol/kg solvent, depending on the specific reaction.

For reactions with excess fatty acid, DCA (0.075 mmol) was mixed with 1.25, 1.5 or 2 mol equivalents of octanoic acid (depending on the specific reaction) in *tert*-butanol (1.5 g).

The reaction vials (one per time-point) were incubated for 1 h at 60 °C, 800 rpm in a shaking thermoblock (Thermal shaker Lite, VWR, Leuven, Belgium) to achieve good pre-mixing of both substrates. Molecular sieves 3 Å (50% in excess compared to the amount needed to absorb all the water that can be formed in the reaction) were then added and the reaction was started by the addition of Novozym^®^435 (1% *w*/*w* solvent). The reactions were incubated at 60 °C, 800 rpm for the specified reaction time (between 15 min and 96 h). Afterwards, the supernatant (leaving out the molecular sieves and lipase beads) was transferred to a new vial and centrifuged for 2 min at 1000 rpm to obtain pellet molecular sieve dust and any remaining lipase beads.

For reactions in solvent-free conditions, DCA was mixed with octanoic acid at a mol ratio acid/alcohol of 2.5, 5 or 10 to obtain a final reaction weight of 0.5 g. The vials (one per reaction time-point) were incubated at 65 °C (melting temperature of DCA), 800 rpm for 1 h before the addition of molecular sieves 3 Å (50% in excess) and lipase (3% *w*/*w* substrates). Reactions were incubated at 65 °C, 800 rpm for the specified reaction time. After reaction, 2 mL of *tert*-butanol was added, and the samples were immediately centrifuged to remove molecular sieves and lipase.

For reactions in solvent-free conditions under a vacuum, DCA was mixed with octanoic acid at a mol ratio acid/alcohol of 10 to obtain a final reaction weight of 6 g. The substrate mix was incubated at 65 °C until DCA was melted. The reaction was started by the addition of lipase (3% *w*/*w* substrates) and application of a vacuum (100 mbar).

All reaction samples were kept at −20 °C until further analysis by UPLC.

### 2.3. Purification and Characterization of DCA-C8

For purification of the ester product dihydroconiferyl octanoate (DCA-C8), the reaction between DCA and octanoic acid was carried out at an equimolar substrate concentration of 50 mmol/kg solvent in 35 g of *tert*-butanol for 24 h at 60 °C. After the reaction, the lipase and molecular sieves were removed by centrifugation and the sample was concentrated by evaporation under a vacuum. The concentrated crude sample was purified by flash column chromatography on silica gel, using petroleum ether/ethyl acetate (90/10 by vol.) as an eluent. Column fractions were analyzed by TLC and those containing the product were pooled and the solvent was evaporated under a vacuum. The end product of dihydroconiferyl octanoate (DCA-C8, a transparent liquid) was analyzed by ^1^H-NMR (Spinsolve 80 MHz ULTRA, Magritek, Aachen, Germany) in CDCl_3_. Data was processed using Mnova software (version 14.3.0, Mestrelab, Santiago de Compostela, Spain). Chemical shifts are expressed in parts per million (ppm) using the chloroform signal as a reference (7.26 ppm). FTIR analysis was carried out using a Thermo Fisher FTIR i10 (Nicolet series) spectrometer (Thermo Fisher Scientific, Geel, Belgium). The measurements were performed in the attenuated reflectance (ATR) mode fitted with a diamond crystal. The spectra were collected in the range of 4000–500 cm^−1^, with a 4 cm^−1^ scan resolution and 32 scans were carried out for every sample. Blank atmospheric scans were subtracted from the sample scans prior to their analysis. Analysis was performed after linear baseline correction and standard normalization (available in Omnic software, v9.9.473) of all the spectral scans.

*4-(3-hydroxypropyl)-2-methoxyphenyl octanoate (DCA-C8)*
*^1^H-NMR (80 MHz, CDCl_3_)*: *δ* 6.79 (m, 3H, Ar); 5.47 (s, 1H, Ar-OH); 4.09 (t, *J =* 6.5 Hz), 2H, -CH_2_-COO-); 3.88 (s, 3H, -OCH_3_); 2.80–2.47 (m, 2H, -CH_2_-); 2.44–2.15 (m, 2H, -CH_2_-); 2.11–1.78 (m, 2H, -CH_2_-); 1.76–0.68 (m, 15H, -CH_2_-). Integration of the signals between 2.80 and 0.68 ppm results in 2 extra H (21 instead of 19), possibly due to the presence of traces of water in CDCl_3_ (NMR solvent) or traces of grease in the petroleum ether (purification solvent).
IR (neat) ν: 3560–3188, 2958–2854, 1733, 1606, 1519, 1363, 1271, 1238, 1155, 1032 cm^−1^.
HRMS in positive mode, [C_18_H_28_O_4_ + NH4]^+^ m/z 326.2324, [C_18_H_28_O_4_ + H]^+^ m/z 309.2058, HRMS in negative mode, [C_18_H_28_O_4_ − H]^−^ m/z 307.19092.

### 2.4. UPLC Analysis

For the quantification of DCA and DCA-C8, the reaction samples were diluted in acetonitrile prior to analysis. The samples (0.5 µL) were injected into an Acquity UPLC system (Waters) coupled to a HClass-DAD detector (Waters) and equipped with a BEH C18 column (particle size 1.7 µm, 2.1 × 100 mm) maintained at 35 °C. Elution was performed at a flow rate of 0.4 mL/min and a pressure of 660 bar, using a gradient of acetonitrile in water from 5% to 95%. Pure DCA and DCA-C8 (synthesized and purified in-house) were used to construct calibration curves for quantification.

### 2.5. Antioxidant Activity Test

The antioxidant capacity of the compounds was determined with the DPPH radical scavenging test according to the guidelines of Sharma et al. [22].

A stock solution of DPPH (100 µM) was prepared in methanol and stored in the dark at 4 °C under N_2_ atmosphere. Stock solutions (0.5 mM) of DCA, PG, BHT and BHA were prepared in methanol and then diluted further in the same solvent to prepare samples in the concentration range of 1–200 µM. For DCA-C8, an initial stock solution (10 mM) was prepared in chloroform, diluted in methanol to form a second stock solution (0.5 mM) and then diluted further to achieve samples in the same concentration range as for the other compounds.

First, a time-course of DPPH scavenging was performed in order to determine the incubation time at which DPPH consumption reaches a plateau for every compound. To achieve this, each compound (final concentration 25 µM) was mixed with a DPPH radical (final concentration 50 µM) and the Abs 517 nm was monitored over 22 h at 20 °C. A standard sample that contained only the DPPH radical (50 µM) was also included to track any natural DPPH decay under test conditions for correction.

For the determination of the IC_50_ of every compound, 1 mL of each concentration (1–200 µM) was mixed with 1 mL of the DPPH stock (100 µM) and incubated at 20 °C for 90 min in the dark. A blank sample (only methanol) and a standard sample (50 µM DPPH in methanol) were also included. DPPH scavenging was monitored by measuring the absorbance at 517 nm.

The DPPH inhibition % (I%) was calculated for every sample concentration with the following formula:

(1) $$I\%=\left(1-\frac{Abs_{sample}-Abs_{blank}}{Abs_{DPPH\;stnd}-Abs_{blank}}\right)\times 100$$

where *Abs_sample_* is the absorption of the sample that contained the antioxidant compound, *Abs_blank_* is the sample with only methanol and *Abs_DPPH stnd_* is the sample that contained only DPPH and no antioxidant compound. The IC_50_ values (µM) of each compound (concentration needed to achieve 50% of DPPH scavenging) were calculated from the linear part of the curve obtained by plotting I% against the compound concentration (µM).

### 2.6. Lignin Depolymerization

Depolymerization of the technical lignins was carried out by heterogenous reductive catalytic hydrogenation in methanol (beechwood lignin) or ethanol (wheat straw lignin), using 5% Pd/C as a catalyst at 235 °C for 2 h, with an initial H_2_ pressure of 30 bar and a reaction pressure of about 60–70 bar. After the reaction, the catalyst was removed by vacuum filtration over celite, the solvent was evaporated in the rotavap and the solid sample was dried under a vacuum at 40 °C. The resulting hydrogenolysis lignin (hereafter referred to as lignin_HL) was further fractionated by extraction with diethyl ether (20 wt% lignin concentration in diethyl ether) into a soluble fraction (30 wt%, hereafter referred to as lignin_HL_Et_2_O soluble) and an insoluble fraction (70 wt%, hereafter referred to as lignin_HL_Et_2_O residue).

### 2.7. Enzymatic Esterification of Lignin

The different lignin fractions (polymeric, HL, HL_Et_2_O soluble and HL_Et_2_O residue) were solubilized in acetone to achieve a concentration of 50 mmol aliphatic OH/L. The fatty acid (octanoic-, lauric- or oleic acid) was added at an equimolar concentration with the aliphatic OHs present in lignin (quantified as described in Section 2.8) and molecular sieves 3 Å (1% *w*/*v*) were used to absorb the water formed during esterification.

The reactions were started by the addition of lipase (3% *w*/*v*) and incubated at 20 °C for 72 h. After incubation, the reactions were centrifuged at 1000 g for 10 min to remove the lipase and molecular sieves and the supernatant was transferred to a new vial. The reaction vials were washed with acetone to ensure that all the substrate was recovered; the washings were combined with the reaction supernatant and acetone was evaporated by N_2_ flushing, followed by vacuum drying at 40 °C.

### 2.8. Characterization of the Lignin Fractions

The molecular weight distribution of the different lignin fractions (before esterification) was determined by gel permeation chromatography (GPC) coupled to UV detection, using THF as a solvent. Molecular weights are given relative to polystyrene standards (162–12,980 g/mol).

The amount of aliphatic and phenolic OH groups in all the lignin fractions (before and after esterification) was quantified by ^31^P-NMR (Spinsolve 80 MHz ULTRA, Magritek, Aachen, Germany) in CDCl_3_/pyridine after sample derivatization with the phosphorylating reagent TMDP, using NHND as a reactive internal standard, as previously described [23]. Data was processed using Mnova software (version 14.3.0, Mestrelab, Santiago de Compostela, Spain). In the samples after esterification, the amount of aliphatic OH expressed as mmol/g sample (including the residual fatty acid from the reaction) was converted to mmol/g lignin, multiplying the value by the wt% of lignin in the sample. The esterification degree was calculated by comparing the aliphatic OH content (expressed as mmol/g lignin) before and after esterification.

FTIR analysis was performed as described in Section 2.3.

## 3. Results and Discussion

### 3.1. DCA Esterification with Different Fatty Acids

**Figure 2:**
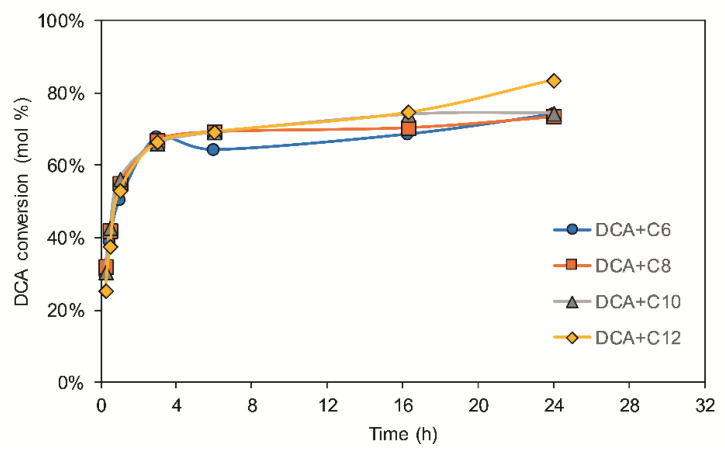
Time-course of DCA esterification with different fatty acids. C6 = hexanoic acid, C8 = octanoic acid (caprylic acid), C10 = decanoic acid (capric acid); C12 = dodecanoic acid (lauric acid).

The enzymatic esterification of DCA, a lignin-derived phenolic alcohol, was performed with different fatty acids with chain lengths ranging from C6 to C12. The lipase could esterify all the fatty acids to the DCA at similar rates, resulting in substrate conversions of 74–84% (mol%) (Figure 2). The conversion rapidly increased during the first 4 h, with values around 30% after 15 min, and then it plateaued, remaining more or less unchanged after 24 h. Incubation for a longer time (up to 96 h) did not result in an increase in substrate conversion. Although the trend of the reaction was similar for all four fatty acids, the final substrate conversions were slightly higher in the case of lauric acid (C12). These results are in line with the literature, since the lipase B from *C. antarctica* (CALB) is reported to be able to esterify fatty acids ranging from C4 to C18, with a higher efficiency for C10–C16 in solvent-assisted reactions [24].

The esterification between DCA and octanoic acid was then chosen as a model for reaction optimization. Octanoic acid, as a mid-chain fatty acid, can increase the lipophilicity of phenolic compounds without the risk of enveloping or burying the aromatic -OHs (which may happen with long acyl chains), thereby limiting their ability to interact with free radicals [25]. In addition, it is already liquid at room temperature, facilitating the solvent-free reaction, and it has a lower boiling point than longer fatty acids, making it easier to remove the unconverted excess from the final product during downstream processing (DSP). The ester product (DCA-C8) was purified by silica column chromatography and its structure and purity confirmed by ^1^H-NMR (Figure S1). The purified DCA-C8 ester was used for the determination of its antioxidant activity and as an analytical standard to calculate the product yields in subsequent reactions.

### 3.2. Antioxidant Activity of DCA-C8

The antioxidant activity of DCA-C8 was measured with the DPPH radical assay and benchmarked against non-esterified DCA and other commercial antioxidants (PG, BHT and BHA). The DPPH assay measures the ability of a compound to scavenge the DPPH radical by H donation and it is one of the most common antioxidant assays used in the literature [26].

Two types of measurements were performed. First, a time-course of DPPH radical scavenging was carried out, using each tested compound at a fixed concentration (25 µM) in order to determine the number of moles of DPPH scavenged per mol of compound after short-term (90 min) and long-term incubation (22 h). Secondly, every compound at concentrations ranging from 0.5 to 100 µM was incubated with a DPPH radical for 90 min in order to calculate the IC_50_ (concentration of antioxidant that results in 50% of the radicals scavenged after a specific incubation time).

**Figure 3:**
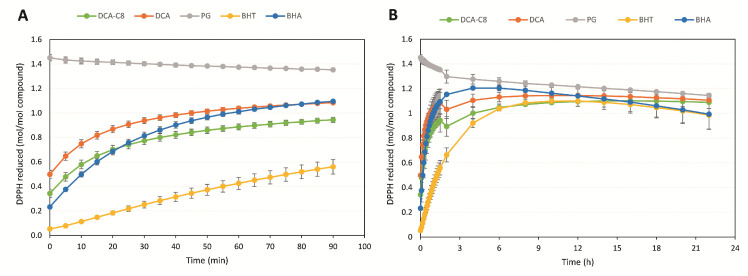
Time-course of DPPH scavenging (**A**) during short-term (90 min) and (**B**) long-term (22 h) incubation. Values are the average of two independent measurements ± standard deviation.

In principle, an antioxidant compound that contains more than one phenolic OH (e.g., PG) should be able to reduce more moles of DPPH radicals per mol of compound, since it can donate more Hs. Antioxidant compounds with only one phenolic -OH (DCA-C8, DCA, BHT and BHA) can only reduce more than one molar equivalent of DPPH if the phenoxy radical formed after donating the H can be somehow regenerated back to a phenolic -OH, enabling them to quench yet another molecule of DPPH. The results of the DPPH scavenging time-course (Figure 3A,B) show that PG reacted very fast with DPPH (completing the reaction within less than 5 min) and was able to reduce more than 1 molar equivalent of the radical (1.4 mol DPPH reduced/mol PG), but not in direct proportion to its number of phenolic -OHs. Moreover, this trend remained relatively unchanged during the long-term incubation with DPPH. DCA-C8 was a slower DPPH quencher than PG, reducing less than 1 mol equivalent of the radical during the first 90 min (short term)_,_ but reaching a 1.1 molar radical equivalent reduction after 22 h (long term). This indicates some regeneration of the phenoxy radical formed. A similar trend was observed for DCA, BHA and BHT, and they all reached the same value of mol DPPH quenched/mol of compound after 22 h as PG (1.1 mol equivalent). The addition of the octyl chain to DCA seemed to have an effect on the antioxidant reaction rate, but the final reduced molar equivalents of DPPH were the same for both non-esterified DCA and DCA-C8.

Lignin model compounds are reported to be able to regenerate phenoxy radicals back to phenolic -OH due to the contribution of methoxy substituents, being able to reduce more than one DPPH molecule per phenolic -OH [27].

**Figure 4:**
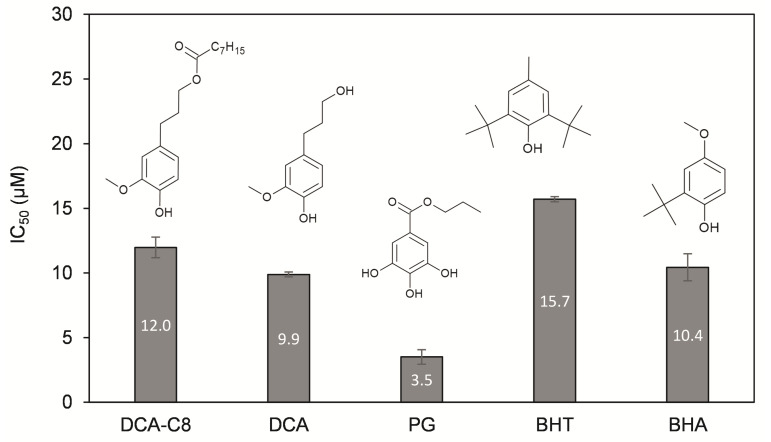
IC_50_ values (90 min) of the ester product DCA-C8 compared to non-esterified DCA and other commercial antioxidants. DCA-C8 = dihydroconiferyl octanoate, DCA = dihydroconiferyl alcohol, PG = propyl gallate, BHT = butylated hydroxytoluene; BHA = butylated hydroxyanisole. Values shown are the average of three independent measurements ± standard deviation. IC_50_ value refers to the concentration of the compound needed to scavenge 50% of the DPPH radical. A lower IC_50_ value means a more potent antioxidant.

The determination of the IC_50_ values (Figure 4) also indicated PG as the fastest antioxidant (IC_50_ = 3.5 µM resulted in 50% DPPH scavenged in 90 min), followed by DCA (9.9 µM) > BHA (10.4 µM) > DCA-C8 (12.0 µM) > BHT (15.7 µM).

The better antioxidant performance of lignin-derived compounds compared to BHT has already been described in the literature [28] and attributed to the stabilizing effect of the ortho methoxy group. Direct comparison with IC_50_ values from the literature cannot be made due to the differences in assay conditions (solvent used for the assay, DPPH concentration, temperature and incubation time).

These results show that the long-term antioxidant activity of DCA-C8 is more relevant than its short term activity; therefore, this compound could be used as an effective antioxidant if time is not a limiting factor in the application.

### 3.3. Optimization of DCA Esterification with Octanoic Acid

**Figure 5:**
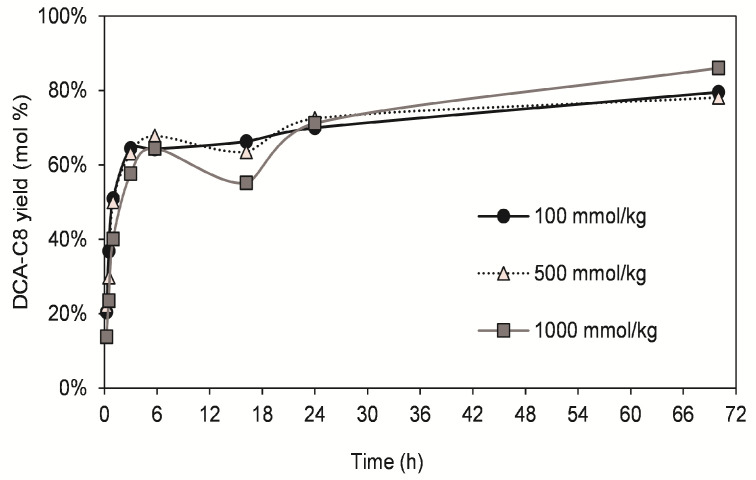
Time-course of DCA-C8 yield in reactions at different equimolar concentrations of DCA and octanoic acid. Reaction conditions: solvent (*tert*-butanol), lipase 1% (*w*/*w* solvent), molecular sieves (1.5× in excess), 60 °C.

The optimization of DCA esterification with octanoic acid was first performed in a solvent (*tert*-butanol) with water removal via molecular sieves and using an equimolar concentration of both substrates (100, 500 or 1000 mmol/kg solvent). The progress of the reaction could be measured in terms of product yield using the DCA-C8 ester synthesized and purified in house. All three concentrations resulted in similar reaction trends, with product yields increasing during the first 6 h and then reaching a plateau (Figure 5). The final product yields were in the range of 79–86% (mol%) after 72 h.

The possibility of increasing product yields by shifting the reaction equilibrium through the addition of one of the substrates in excess (in this case the acid, since it has the lowest boiling point of the two) was also tested using three different molar ratios of octanoic acid/DCA (1.25, 1.5 and 2) in a solvent-assisted reaction (Table 1). The addition of 2 molar equivalents of octanoic acid with respect to DCA resulted in the almost complete conversion of DCA to an ester product after 24 h (97.1% yield).

Next, the possibility of performing the reaction in solvent-free conditions was explored. Solvent-free reactions offer several advantages, such as avoiding the use of organic solvents, producing higher substrate and product loads and the simplification of DSP. For a solvent-free reaction to be feasible, at least one of the substrates needs to be liquid under the reaction conditions to ensure good mixing. In the case of DCA esterification with octanoic acid, both substrates are liquid if the reaction temperature is increased to 65 °C (matching the melting point of DCA). The solvent-free reaction was first carried out in the presence of desiccants (molecular sieves) for water removal, using three different molar ratios of octanoic acid/DCA (2.5, 5 and 10). In all three cases, maximum product yields were obtained after 3 h (Table 1) and close to maximum yields were already achieved after 30 min (data not shown). This shows the extent to which the reaction can benefit from solvent-free conditions regarding the esterification rate. The highest product yield (97.5%) was achieved when octanoic acid was 10x in molar excess with respect to DCA, although the use of less acid (5× in excess) also resulted in a satisfactory yield (93.6%). Octanoic acid added in only 2.5 molar equivalents with respect to DCA gave a lower product yield (88.5%). The product DCA-C8 was also formed in significant amounts (20–33% yield) in the control reactions where no lipase was added, but only after 24 h (Table 1). In solvent-free conditions, the contact between substrates is maximized, and the use of excess acid (which can autocatalyze the esterification process to some extent) and high temperatures enable the reaction to take place even without a catalyst. However, product yields will remain low if water is not removed to shift the equilibrium, and the addition of a catalyst (in this case lipase) is needed to increase the reaction rate and to ensure that esterification takes place only on the aliphatic OH groups.

The solvent-free reaction was also performed using a vacuum for water removal instead of desiccants, and a molar ratio of octanoic acid/DCA = 10. Under these conditions, the product yield reached 90.3% after 3 h (Table 1), which was lower than in the solvent-free reaction with desiccants. The product yield of the control reaction without lipase was also lower (17.3% after 24 h).

These results show that adding an excess of fatty acid in the reaction is a good strategy to shift the equilibrium towards the product, but this also entails the removal of unconverted substrate at the end of the process. In this case, the solvent-assisted reaction required a lower fatty acid excess in order to achieve the almost complete DCA conversion compared to the solvent-free condition. However, even though *tert*-butanol is considered as a green solvent [29] and can be easily removed from the product via evaporation under a vacuum, its use brings additional hazards to the process due to its volatility and flammability. On the other hand, the solvent-free reaction avoids these hazards and aids in achieving high product yields in significantly shorter times. The choice of one reaction strategy or the other should be based on a careful analysis that takes into account all aspects of the process, such as energy use, reaction time, reagent/product costs, downstream processing, safety/environmental hazards, etc.

The use of lipases to catalyze the selective esterification of aliphatic -OHs in phenolic substrates (other than DCA) with the purpose of producing lipophilic antioxidants has been reported in the literature before. For example, Buisman et al. esterified hydroxytyrosol with 1 molar equivalent of octanoic acid using immobilized CALB, achieving 70–85% product yields in solvents such as diethyl ether, pentane and hexane [30]. These yields are similar to those obtained in this present work for DCA in the solvent reaction at equimolar substrate concentrations.

Torres de Pinedo et al. used the same lipase Novozym 435 to esterify several phenolic alcohols that varied in side chain length with butyric-, palmitic- and stearic acid [20]. When they performed the reaction in acetonitrile with 2 molar equivalents of the free fatty acid, the product yields were 69–85% (depending on the type of phenolic alcohol), which are lower than those obtained in this work in the solvent-assisted reaction with excess fatty acid (97.5% after 24 h). When the reaction was carried out under solvent-free conditions using the ethyl ester of the fatty acid as a substrate and as a solvent, they could increase product yields up to 98% (after 6–16 h, depending on the phenolic alcohol), but only when the acyl donor was used in high excess (30 molar equivalents). In our work, the solvent-free reaction resulted in a similar yield (97.5% after 3 h) with the advantage that the free fatty acid was used, and in lower excess (10 molar equivalents).

### 3.4. Enzymatic Esterification of Lignin

As a next step, we performed the lipase-catalyzed esterification of various lignin fractions from different botanical origins, including beechwood (hardwood) or wheat straw (grass). The original technical lignins were subjected to depolymerization by reductive catalytic hydrogenation in methanol or ethanol using Pd/C as a catalyst. The compounds in the depolymerized lignin samples were further fractionated with diethyl ether to obtain a soluble and an insoluble fraction. All the lignin fractions were characterized by ^31^P-NMR and GPC to determine their aliphatic and phenolic OH content and their Mw distribution (Table 2). Beechwood lignin and its hydrogenolyzed fraction contain a similar amount of aliphatic OH to the wheat straw lignin fractions, but a higher phenolic OH content.

All the lignin samples were used as a substrate for enzymatic esterification, except the technical wheat straw lignin, which was not soluble in the reaction solvent acetone. The lignin concentration in the reaction was expressed as mmol/L of aliphatic OH, since the lipase selectively esterifies these groups, and the fatty acid (octanoic, lauric or oleic) was added in 1 mol equiv. to the aliphatic -OHs.

**Figure 6:**
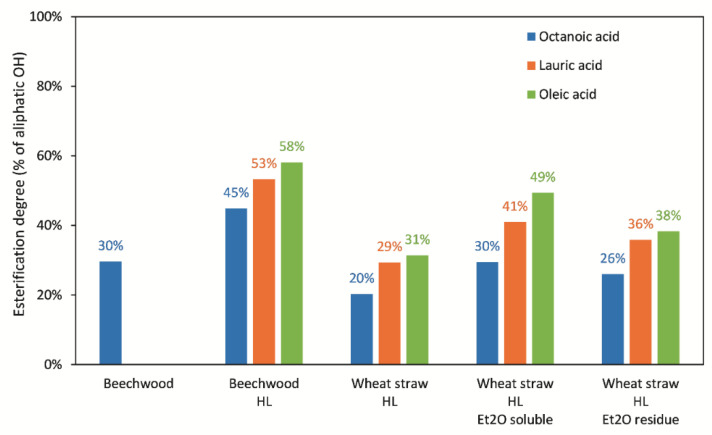
Esterification degree of the different lignin fractions with various fatty acids. The reactions were performed using 1 molar equivalent of fatty acid with respect to the aliphatic OH groups in lignin, using acetone as a solvent, and catalyzed by lipase at 20 °C for 72 h. The amount of remaining free aliphatic OH groups after esterification was quantified by ^31^P-NMR in every reaction as mmol/g sample (including the residual fatty acid) and it was converted to mmol/g of lignin by multiplying the value with the mass % of lignin in the sample. The corrected value was then compared to the aliphatic OH content (mmol/g lignin) in the original lignin fractions in order to calculate the esterification degree.

The results of the esterification degree for every combination of lignin fraction and fatty acid are shown in Figure 6. For all the lignin fractions, the esterification degree increased with the length of the fatty acid used as an acyl donor, which is consistent with the reported chain-length preference of this lipase [24]. The enzyme esterified depolymerized beechwood lignin to a higher extent than depolymerized wheat straw lignin, possibly due to the presence of more free primary aliphatic OH groups in the former. Wheat straw lignin is known to have some of its primary OH (*ϒ*-OH) groups esterified with acetate or *p*-coumarate [31].

When comparing the results from the three depolymerized wheat straw lignin fractions, it seems that the lipase could achieve a higher esterification degree in the fraction that contained compounds of lower Mw (HL_Et_2_O soluble), most likely due to the better accessibility of these compounds to the active site of the enzyme.

By the ^31^P-NMR analysis method used in this work, it is not possible to distinguish the type of aliphatic OH (primary or secondary) [32]; therefore, a direct comparison of the esterification degree without this information is not entirely accurate, since the lipase can only esterify the primary groups.

FTIR analysis was also used to confirm that the reaction had taken place. The esterified lignin samples showed a decrease in the signal at 1730 cm^−1^ (corresponding to the disappearance of the fatty acid) and a broadening of the signal between 1700 and 1750 cm^−1^ (corresponding to the formation of the ester linkage), compared to lignin samples from a control reaction (without enzymes) (Figure S2).

Enzymatic esterification of lignin model compounds has been reported in the literature and confirms the selectivity of the lipase for primary aliphatic OH groups in the presence of phenolic OHs [18,33]. However, the use of lipases to enzymatically esterify real lignin or lignin fractions is scarcely reported in the literature and mainly focuses on polymer applications. For example, lipase-catalyzed reactions have been reported for the esterification of oleate chains into polymeric kraft lignin, in order to increase its compatibility with polyolefins and re-valorizing it into composites [34]. The reaction was performed in ionic liquids to overcome the low solubility of polymeric lignin and increase its accessibility as a substrate for the enzyme. The maximum esterification yield achieved was 27% based on acyl donor consumption. In another study, Vibha and Negi used a lipase to esterify adipic acid into lignols derived from enzymatically depolymerized lignin. The esterified products were then reacted with styrene to synthesize biopolymers with high thermostability and antioxidant capacity [35].

The chemical esterification of lignin fractions with the purpose of producing antioxidant additives that are miscible with hydrophobic matrixes has recently been reported [9]. In this case, several lignin samples, including low Mw fractions derived from RCF, were esterified with palmitoyl chloride to make them compatible with castor oil (biolubricant). Even though the authors selected pyridine as a catalyst due to its reported preference for aliphatic OHs, the esterification of phenolic OHs was still inevitable. As a consequence, the esterified lignin fractions showed a decrease in their antioxidant activity. The best antioxidant performance (both before and after esterification) was obtained for the fraction with the lowest Mw and highest content of siringyl and guaiacyl units (with radical-stabilizing methoxy groups). This shows that lignin depolymerization allows the production of efficient, bio-based antioxidant additives, but a more selective esterification method is needed in order to tailor their hydrophobicity to the application without compromising their antioxidant capacity. Due to the low cytotoxicity of lignin [36], these lignin-based lipophilic antioxidants could be suitable for applications in personal care products.

## 4. Conclusions

In this work, lignin-derived phenol DCA was selectively esterified at the aliphatic OH with octanoic acid via a lipase-catalyzed reaction. The resulting ester product of DCA-C8 showed relevant long-term antioxidant activity, comparable to other commercial petroleum-based antioxidants, and could be used for applications in which the additional acyl chain renders the antioxidant more lipophilic and suitable for application in hydrophobic matrixes (e.g., in cosmetics). The addition of the fatty acid to the reaction in molar excess allowed the almost complete conversion of DCA into an ester product, both under solvent-assisted and solvent-free conditions. The lipase was also successfully applied to selectively esterify the aliphatic OHs in different depolymerized lignin fractions obtained by an heterogeneous catalytic reductive process. As a logical next step, the esterified lignin fractions should be fully characterized regarding Mw distribution and functional group composition and compared for their antioxidant properties in order to determine the structure–activity relationship.

This study is in agreement with the current needs for the development of innovative, environmentally friendly and biobased alternatives to classical synthetic ingredients in personal care. Increasing the hydrophobicity of lignin via selective esterification is a step forward in utilizing the functional and bioactive properties of lignin.

## Figures and Tables

**Table 1:** DCA-C8 yields in different reactions with an excess of octanoic acid, both in solvent and solvent-free conditions. The C8/DCA values displayed correspond to the molar ratio between the two substrates.

Type of Reaction	DCA-C8 Yield (mol%) (Hours)
^a^ In solvent + desiccants	
C8/DCA = 1.25	64.9% (3 h), 83.0% (24 h)
C8/DCA = 1.5	71.9% (3 h), 84.6% (24 h)
C8/DCA = 2	78.7% (3 h), 97.1% (24 h)
^b^ Solvent-free + desiccants	
C8/DCA = 2.5	88.5% (3 h), 86.2% (24 h)
C8/DCA = 5	93.6% (3 h), 89.3% (24 h)
C8/DCA = 10	97.5% (3 h), 95.1% (24 h)
*No lipase, C8/DCA = 2.5*	20.1% (24 h)
*No lipase, C8/DCA = 5*	28.3% (24 h)
*No lipase, C8/DCA = 10*	33.6% (24 h)
^c^ Solvent-free + vacuum	
C8/DCA = 10	90.3% (3 h), 89.5% (24 h)
*No lipase, C8/DCA = 10*	17.3% (24 h)

^a^ Reaction conditions: DCA (50 mmol/kg), lipase 1% (*w*/*w* solvent), molecular sieves 3× in excess, 65 °C. ^b^ Reaction conditions: reaction scale 0.5 g, lipase 3% (*w*/*w* substrates), molecular sieves 1.5× in excess, 65 °C. ^c^ Reaction conditions: reaction scale 6 g, lipase 3% (*w*/*w* substrates), vacuum 100 mbar, 65 °C.

**Table 2:** Structural characterization of the different lignin fractions by ^31^P-NMR.

	Aliphatic OH	Phenolic OH	COOH	Mw	Mn	Ɖ
Lignin	(mmol/g)	(mmol/g)	(mmol/g)	(Da)	(Da)	Mw/Mn
Beechwood	2.32	4.09	0.10	2210	1010	2.19
Beechwood_HL	2.32	5.01	0.09	1590	874	1.82
Wheat straw	2.31	1.51	0.71	3200	1050	3.0
Wheat straw_HL	1.94	2.87	0.31	1510	744	2.03
Wheat straw_HL_Et_2_O_soluble	1.41	2.28	0.23	593	438	1.35
Wheat straw_HL_Et_2_O_residue	2.31	2.93	0.20	1920	1000	1.92

## Data Availability

The data that support the findings of this study are available from the corresponding author (WD) upon reasonable request.

## Supplementary Materials

The following supporting information can be downloaded at: https://www.mdpi.com/article/10.3390/antiox12030657/s1, Figure S1: Annotated ^1^H-NMR spectrum of DCA-C8 (CDCl_3_, 80 MHz). The signals in the 0.5–2.75 ppm region (in blue) correspond to the -CH_2_- of the octanoate chain from the fatty acid and the propanoid chain of DCA. Integration of these signals results in 2 extra H (21 instead of 19) maybe due to the presence of traces of water in CDCl_3_ (NMR solvent) or traces of grease in petroleum ether (purification solvent); Figure S2: FTIR spectrum of DCA-C8; Figure S3: HRMS spectra of DCA-C8 in positive and negative mode; Figure S4: FTIR spectra of esterified lignin fractions compared to lignin fractions from control reactions (without enzyme addition). A decrease in the signal at 1730 cm^−1^ corresponds to the disappearance of the fatty acid and a broadening of the signal between 1700 and 1750 cm^−1^ corresponds to the formation of the ester linkage.
