# Supplementary material for: Selective Enzymatic Esterification of Lignin-Derived Phenolics for the Synthesis of Lipophilic Antioxidants
Source: Antioxidants (Basel). 2023 Mar 7;12(3):657. doi: 10.3390/antiox12030657 (PMC10045519; doi:10.3390/antiox12030657)

MS pos

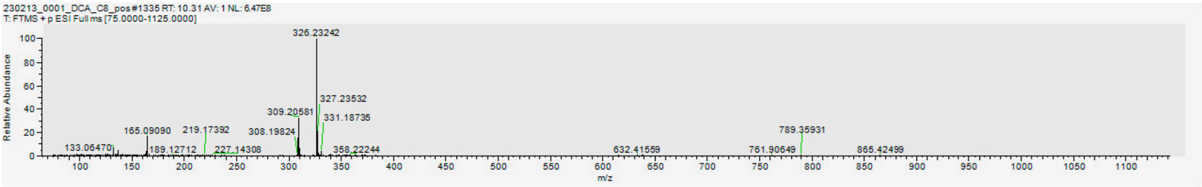

Peak Mass	Display Formula	S Fit	RDB	Delta [ppm]	Theo. mass
326.23242	$C_{18}H_{28}O_4$ NH_4+	46.04	3.50	-0.50	326.23258
Peak Mass	Display Formula	S Fit	RDB	Delta [ppm]	Theo. mass
309.20578	$C_{18}H_{28}O_4$ H+	45.3	4.50	-0.83	309.20604

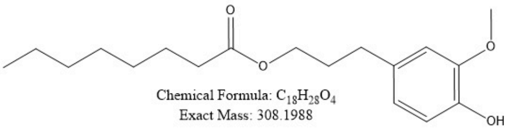

MSMS pos

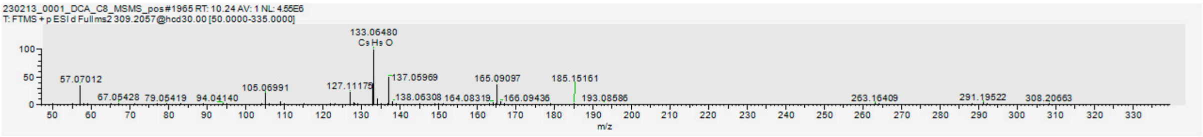

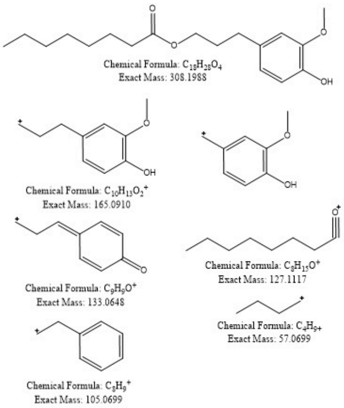

MS Neg

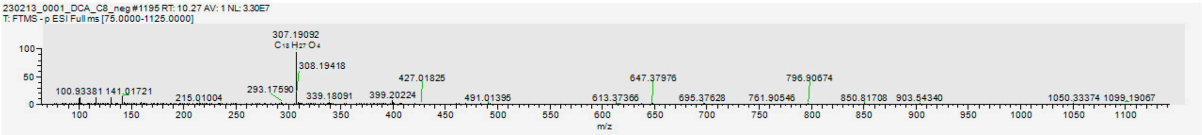

Peak Mass	Display Formula	S Fit	RDB	Delta [ppm]	Theo. mass
307.19092	$C_{18}H_{28}O_4$ H-	70.0	5.50	1.73	307.19039

MSMS neg

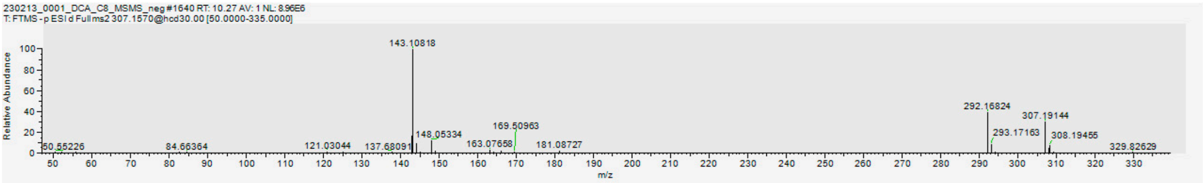

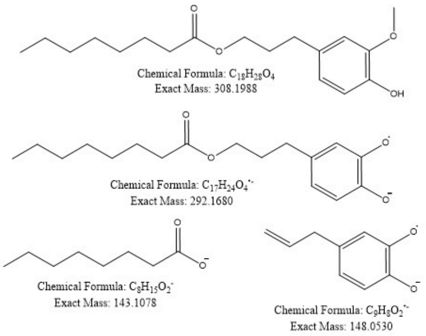

Supplement: Supplementary file 1 [file antioxidants-12-00657-s001.zip › Fig S3 - HRMS spectra of DCA-C8.pdf]
