# Supplementary material for: Selective Enzymatic Esterification of Lignin-Derived Phenolics for the Synthesis of Lipophilic Antioxidants
Source: Antioxidants (Basel). 2023 Mar 7;12(3):657. doi: 10.3390/antiox12030657 (PMC10045519; doi:10.3390/antiox12030657)
Supplement: Supplementary file 1 [file antioxidants-12-00657-s001.zip › Supplementary figures captions.pdf]

Figure S1. Annotated ^{1}H-NMR spectrum of DCA-C8 ($CDCl_3$, 80 MHz). The signals in the 0.5 – 2.75 ppm region (in blue) correspond to the -CH_2- of the octanoate chain from the fatty acid and the propanoid chain of DCA. Integration of these signals results in 2 extra H (21 instead of 19) maybe due to the presence of traces of water in $CDCl_3$ (NMR solvent) or traces of grease in petroleum ether (purification solvent).

Figure S2. FTIR spectrum of DCA-C8.

Figure S3. HRMS spectra of DCA-C8 in positive and negative mode.

Figure S4. FTIR spectra of esterified lignin fractions compared to lignin fractions from control reactions (without enzyme addition). A decrease in the signal at 1730 cm^{-1} corresponds to the disappearance of the fatty acid and a broadening of the signal between 1700 and 1750 cm^{-1} corresponds to the formation of the ester linkage.
