# Supplementary figures and images for: Selective Enzymatic Esterification of Lignin-Derived Phenolics for the Synthesis of Lipophilic Antioxidants
Source: Antioxidants (Basel). 2023 Mar 7;12(3):657. doi: 10.3390/antiox12030657 (PMC10045519; doi:10.3390/antiox12030657)

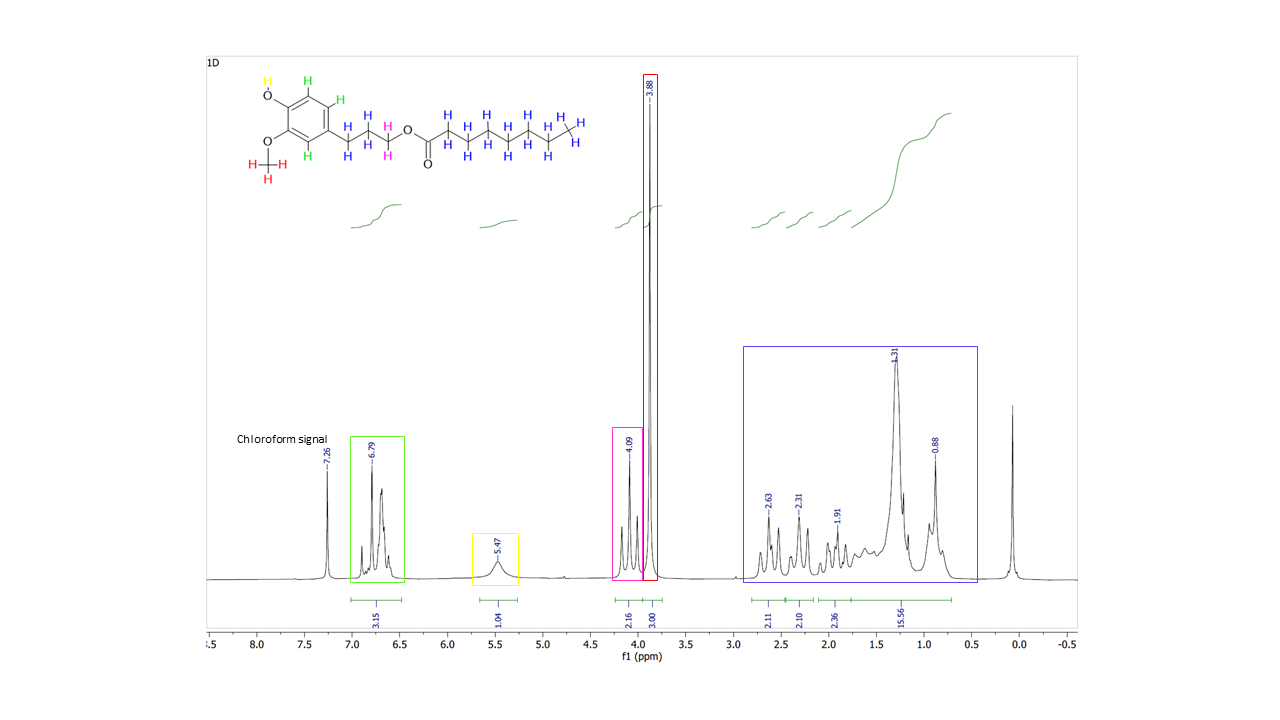

Supplement: Supplementary file 1 [file antioxidants-12-00657-s001.zip › Fig S1- DCA-C8 -NMR spectrum (anotated).tif]

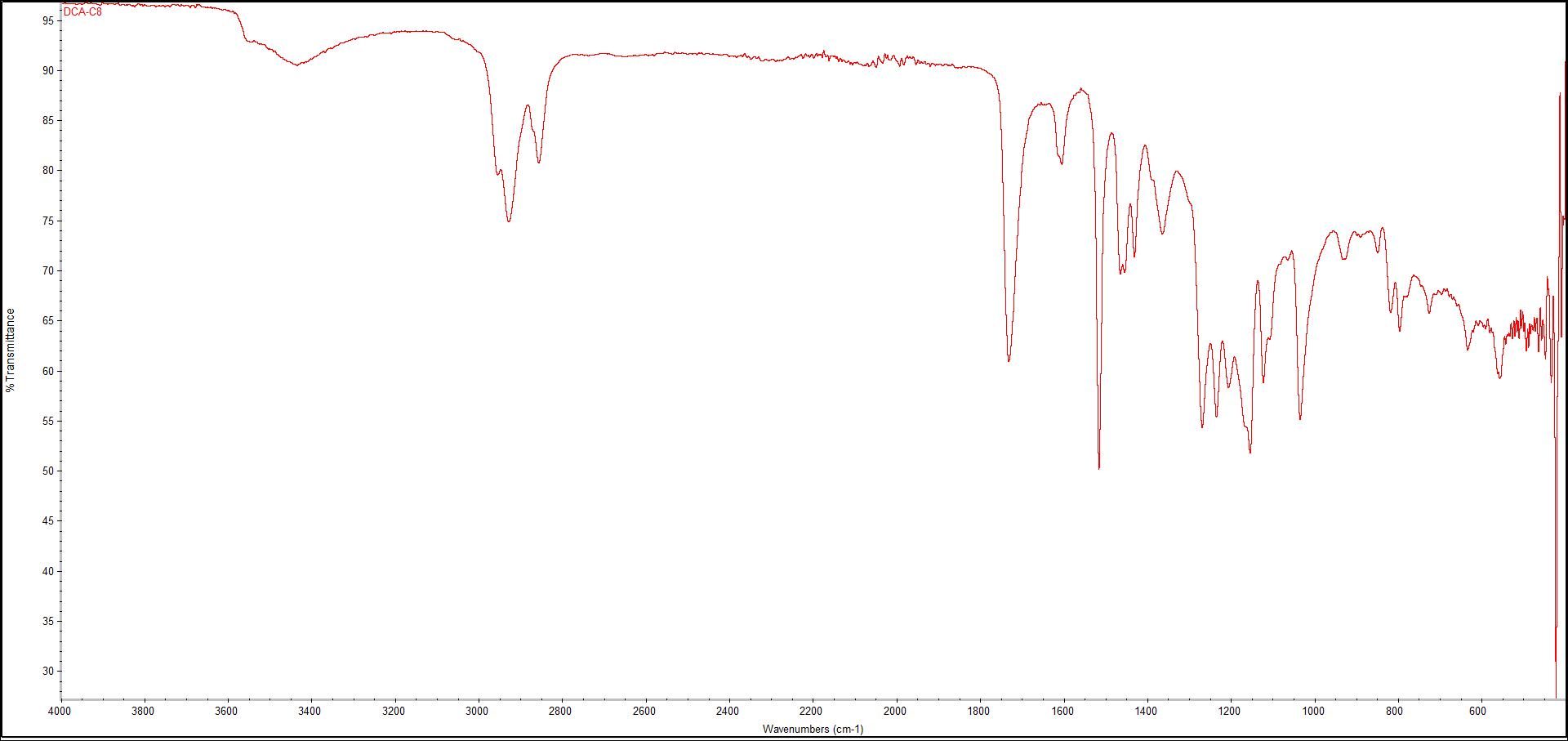

Supplement: Supplementary file 1 [file antioxidants-12-00657-s001.zip › Fig S2 - FTIR spectrum of DCA-C8.TIF]

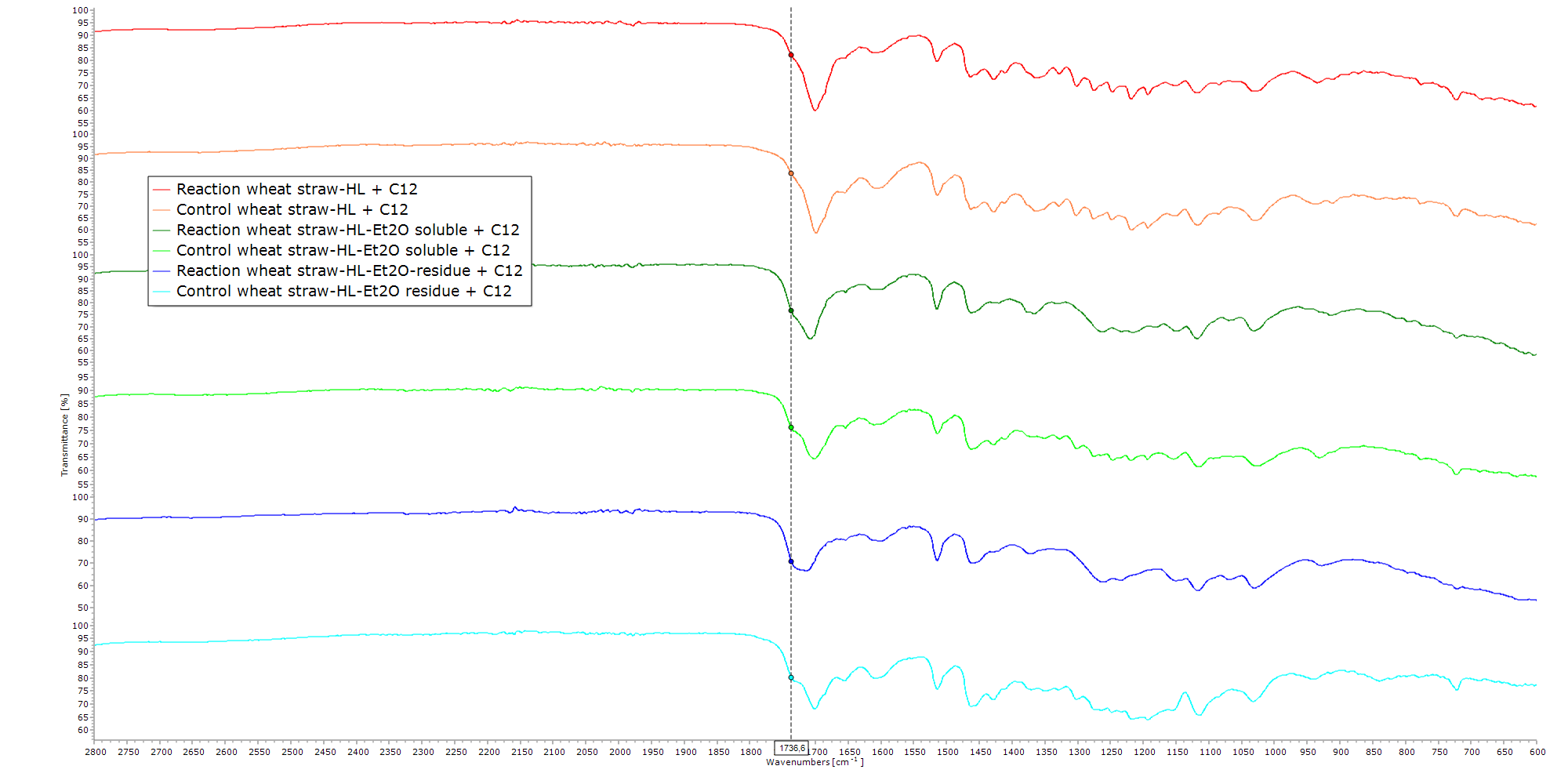

Supplement: Supplementary file 1 [file antioxidants-12-00657-s001.zip › Fig S4 - FTIR spectra lignin reactions.png]
